# Impairments in peroneal muscle size and activation in individuals with patellofemoral pain in weight‐bearing position

**DOI:** 10.1002/jfa2.12014

**Published:** 2024-05-21

**Authors:** Abbis Jaffri, Amber Schwarting, Andrea Baellow

**Affiliations:** ^1^ Department of Physical Therapy Creighton University Omaha Nebraska USA; ^2^ Department of Kinesiology University of Virginia Charlottesville Virginia USA

**Keywords:** anterior knee pain, chronic pain, fibularis muscles, foot posture, rehabilitation

## Abstract

**Background:**

Patellofemoral pain (PFP) is characterized by chronic pain in the anterior aspect of the knee during loading activities. Many studies investigating muscle morphology changes for individuals with PFP focus on the proximal joints, however, few studies have investigated muscles of the foot and ankle complex. This study aimed to explore the differences in peroneal muscle size and activation between individuals with PFP and healthy controls using ultrasound imaging in weight‐bearing.

**Methods:**

A case‐control study in a university lab setting was conducted. Thirty individuals with PFP (age: 20.23 ± 3.30 years, mass: 74.70 ± 27.63 kgs, height: 161.32 ± 11.72 cm) and 30 healthy individuals (age: 20.33 ± 3.37 years, mass: 64.02 ± 11.00 kgs, height: 169.31 ± 9.30 cm) participated. Cross‐sectional area (CSA) images of the peroneal muscles were taken in non‐weight bearing and weight‐bearing positions. The functional activation ratio from lying to single‐leg standing (SLS) was calculated.

**Results:**

There was a statistically significant (*p* = 0.041) group (PFP, healthy) by position (non‐weight‐bearing, weight‐bearing) interaction for the peroneal muscle CSA with a Cohen's d effect size of 0.2 in non‐weight‐bearing position and 0.7 in weight‐bearing position. The functional activation ratio for the healthy group was significantly more (*p* = 0.01) than the PFP group.

**Conclusion:**

Peroneal muscles were found to be smaller in size in those with PFP compared to the healthy subjects in the weight‐bearing SLS position. This study found that those with PFP have lower activation of peroneal muscles in functional position.

## 1 INTRODUCTION

Patellofemoral pain (PFP) is characterized as chronic pain (in absence of other diagnosed pathologies such as tendonitis, chondromalacia patellae, etc.) in the anterior aspect of the knee joint while performing loading activities such as squatting, ambulating, jumping, and jogging, etc. [1]. In their lifetimes, 23% of adults will get PFP; 50% of them will experience PFP that lasts up to 20 years [2, 3]. Because of the chronicity of pain, patients with PFP decrease their daily physical activity, which could lead to cardiovascular diseases and early onset osteoarthritis of the patellofemoral joint [4]. Although many people are affected by PFP, clinical treatment guidelines to treat PFP are still in the developing stage because of the lack of consensus about PFP's underlying etiology [5]. Poor lower‐limb mechanics (hip adduction, greater knee internal rotation, and foot pronation) associated with muscular dysfunction has been found in individuals with PFP [6]. Most of the studies investigating changes in muscle morphology focus specifically on the proximal joints (knee and hip); very few have assessed the muscles of distal joints, such as the foot and ankle complex [7, 8].

The poor limb alignment in the PFP group is not limited to proximal joints only; it is also observed in distal (foot and ankle) joints, which warrants investigation of foot and ankle musculature in individuals with PFP [6]. Nevertheless, a recent study investigated the morphology of ankle and foot muscles, such as intrinsic foot muscles, in a PFP group and found significant atrophy of the abductor hallucis compared to healthy controls [9]. Similarly, peroneal muscles (alternatively referred as fibularis) are another less studied group of muscles in individuals with PFP [10]. A recent meta‐analysis only reported two studies that investigated peroneal changes with conflicting results [10]. Moreover, it is believed that persistent need to control foot mobility during weight‐bearing tasks may result in morphological changes in the foot and ankle muscles, including the peroneal muscles, which act as forefoot stabilizers during heel rise [11]. This may also contribute to the collapse of arches during more dynamic activities, thereby exacerbating the dynamic valgus of the knee associated with PFP. However, previously, most of the assessments of muscle morphology of lower extremity muscles have been performed in non‐weight bearing positions, which are not functional positions for lower extremities and may not show any morphological changes in these muscles [9]. It is of critical importance to assess the morphology and activation of peroneal muscles in the weight‐bearing functional position. The portable nature of ultrasound imaging (USI) provides a unique avenue to assess these muscles in the functional weight‐bearing position [9]. To our knowledge, no previous study has assessed the muscle size and activation of peroneal muscles in the weight‐bearing functional position in the PFP group. Therefore, the purpose of this study is to investigate the muscle size and activation of peroneal muscles in a weight‐bearing functional position in a PFP group and healthy controls. We hypothesize that the PFP group will behave differently than the healthy group in the weight‐bearing position with lower activation of peroneal muscle groups as compared to healthy controls.

## 2 METHODS

This was a case‐control study in which the independent variables were groups (PFP and healthy) and positions (non‐weight‐bearing side lying and weight‐bearing single‐leg standing (SLS) position). The dependent variables were peroneal size (cross‐sectional area (CSA) of peroneal muscles) and functional activation ratio (FAR) assessed using USI.

### 2.1 Participants

Sixty participants (30 healthy, 30 PFP) between the age of 18–37 were included in this study. Demographic information is provided in Table 1. Institutional Review Board approval was obtained for this project and all participants provided written consent prior to data collection. The participants were recruited in the “healthy” group if they had no previous lower extremity surgery, no history of ankle sprains, no lower extremity injury in the 6 months prior to enrollment, and no known neurological dysfunction. PFP participants had additional inclusion criteria based on previous literature [12]. PFP participants were included if they had insidious symptom onset without history of traumatic event, persistent pain for more than 3 months, and presence of retro‐patellar knee pain while performing at least two to of the following activities: stair ascent or descent, kneeling, squatting, running, prolonged sitting, isometric quadriceps contraction, jumping, or pain on palpation of the lateral or medial aspect of patella. In addition, participants with PFP had to score 85 or lower on the Anterior Knee Pain Scale (AKPS) [12] and a score of 3.0 cm or more on a 10 cm visual analog scale (VAS) for the worst pain in the last 72 h before testing (Table 1) [12]. Participants were excluded if they had any other ligamentous injury in the knee, instability, or any other source of anterior knee pain.

**TABLE 1:** Participants demographics.

	PFP (*n* = 30)	Healthy (*n* = 30)	*p*‐value
Sex	19 F, 11 M	19 F, 11 M	
Age (yrs)	20.23 ± 3.30	20.33 ± 3.37	0.90
Height (cm)	161.32 ± 11.72	169.31 ± 9.30	0.46
Weight (kg)	74.7 ± 27.630	64.02 ± 11.00	0.06
Duration of pain (mo)	46.7 ± 28.32	0 ± 0	<0.01
Current VAS	2.01 ± 1.64	0 ± 0	<0.01
Worst VAS 72 h	4.70 ± 1.42	0 ± 0	<0.01
AKPS	77.13 ± 9.34	100 ± 0	<0.01
FPI	2.46 ± 3.93	1.9 ± 2.74	0.52

Abbreviations: AKPS, Anterior Knee Pain Scale; cm, centimeters; FPI, Foot Posture Index; kg, kilograms; mo, months; PFP, patellofemoral pain; VAS, visual analog scale; yrs, years.

### 2.2 Procedures

After consenting and enrolling based on eligibility criteria, participants' demographics (age, weight, height, and duration of pain) were collected. Following demographic information, foot posture evaluation and USI imaging was performed. Foot posture was assessed by a physical therapist with 5 years of clinical experience using Foot Posture Index (FPI). FPI analysis has been previously described in detail [13]. Briefly, FPI is scored on a five‐point scale, with scores ranging from −12 to +12. The higher the score, the more pronated the foot posture [13].

**FIGURE 1:**
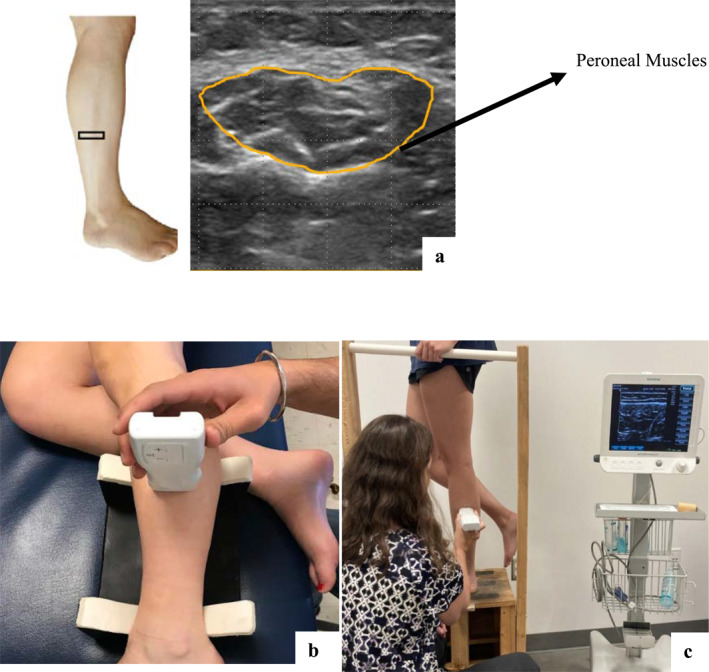
Ultrasound imaging procedure in prone and unipedal single‐leg standing position. (a) Ultrasound linear transducer placement and CSA measurement for assessment of peroneal muscles. The probe is placed at 50% of the fibular length. This percentage was calculated as a midpoint between the fibular head and the lateral malleolus. The yellow line shows the CSA measurement performed for the peroneal muscles alongside the fascial borders. (b) Represents the non‐weight‐bearing prone position that was used for imaging peroneal muscles in the non‐weight‐bearing position. (c) Represents the weight‐bearing bipedal position that was used for imaging peroneal muscles in the weight‐bearing position. CSA, cross‐sectional area.

For USI, a Siemens Acuson Freestyle US system with a wireless 8‐MHz linear transducer (Siemens, Mountain View) was used. All the USI was performed at a depth of 3.5 cm. The USI was performed in both non‐weight‐bearing side lying position [14] (Figure 1b) and weight‐bearing SLS position [9] (Figure 1c). Three images were taken and averaged for each position. Previously established reliable methods by Angin et al. [15] were used for probe placement which was 50% of the fibular length (Figure 1a). Participants stood on the leg that was imaged. For the healthy group, the right leg was imaged for all participants; the ipsilateral leg with the worst knee pain was imaged in the PFP group. Three images were taken for peroneal muscles in both non‐weight‐bearing sidelying and the weight‐bearing SLS functional position.

### 2.3 Data processing

Although all the images were taken by the same investigator with 4 years of experience in performing US imaging, intra‐observer reliability was assessed using intraclass correlation coefficient (ICC) for the images taken. Three images were measured in both positions for muscle size; averages were taken for each measure. CSA in cm^2^ was assessed using the freehand tool in ImageJ. For normalization of the size measures, the CSA in cm^2^ was divided by body mass in kgs [9]. Functional Activation Ratios (FAR) also were calculated in this study; these are calculated by taking a ratio of CSA measures in the functional task divided by CSA measures in the resting state [14, 16].

**Ratio**: CSA (weight‐bearing position).

CSA (non‐weight‐bearing position).

### 2.4 Statistical analysis

Statistical analysis was performed using IBM Statistics (v26.0, SPSS, Inc.). Normalization, skewness, and kurtosis of the dependent variables were assessed. ICC was used to assess the intra‐rater reliability for the peroneal muscle CSA images taken [17]. Independent‐t test was used to assess the differences in group demographics (age, weight, height, and duration of pain) and patient‐reported questionnaires (AKPS and VAS). A 2 × 2 repeated‐measure split‐plot ANOVA was used to analyze the muscle size measure for the two groups and between two positions (non‐weight‐bearing sidelying position and weight‐bearing SLS position). An independent *t*‐test was used to assess the differences between the two groups for the FAR. Pearson correlation analysis was used to assess the relationship between FPI and weight‐bearing peroneal muscle CSA. Classification was set a priori as 0.0–0.4 (weak), 0.4–0.7 (moderate), and 0.7–1.0 (strong) [18]. An a priori *α* level of <0.05 was used for all analyses. Cohen's d effect size with 95% confidence interval (CI) was used to analyze the magnitude of difference in peroneal muscle CSA. The strength of effect size is categorized as trivial = 0–0.2, small = 0.21–0.5, moderate = 0.51–0.8, and large > 0.8 [19].

## 3 RESULTS

Dependent variables of interest were all normally distributed based on skewness, kurtosis, and normality of data assessed using Levene's test (*p* > 0.05). There was no statistically significant difference (*p* > 0.05) in height, mass, and age between the two groups (Table 1). Details of demographics are provided in Table 1. The peroneal muscles CSA images exhibited excellent intra‐rater reliability (ICC = 0.95–0.99).

**FIGURE 2:**
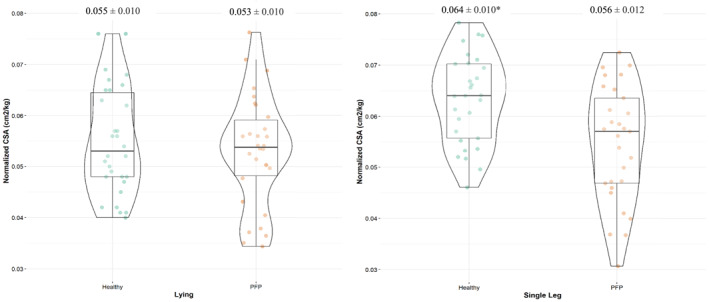
Peroneal cross‐sectional area in PFP and Healthy Individuals. Green dots represent individual participants from the healthy group; yellow dots represent individual participants from the PFP group. Group means ± SD (cm^2^/kg) are provided at the top of each group. * Represents statistical significance. PFP, patellofemoral pain.

A statistically significant (*p* = 0.041) interaction was observed between the groups (PFP, healthy) and positions (non‐weight‐bearing, weight‐bearing) for the peroneal muscle CSA. Specifically, the CSA was found to be smaller in the PFP group, particularly in the weight‐bearing SLS position (Figure 2). The effect size for the peroneal muscle CSA between the PFP and healthy group for the non‐weight‐bearing position was 0.20; for the weight‐bearing SLS position, it was 0.70. FAR for the healthy group (1.21 ± 0.29) was significantly more (*p* = 0.01) than the PFP group (1.06 ± 0.17). We also found a statistically significant (*p* = 0.001) Pearson correlation of *r* = −0.52 between the FPI and Peroneal muscle size CSA for the PFP group in the SLS position.

## 4 DISCUSSION

To our knowledge, this is the first study to investigate the morphological changes in peroneal muscles in non‐weight‐bearing and weight‐bearing positions in the PFP group. A significant interaction between groups (PFP and healthy) and positions (non‐weight‐bearing and weight‐bearing) for peroneal CSA indicates that the CSA increased differently for the two groups in both positions. There was a greater increase in the CSA of peroneal muscles in the SLS position for the healthy group compared to the PFP group. Similarly, it was observed that FAR was higher in the healthy group compared to the PFP group, showing greater activation of these peroneal muscles in a more functional SLS position [16]. We also identified a significant inverse correlation between the FPI score and the peroneal muscle size in the SLS position. This suggests that as the foot becomes more pronated, the peroneal muscle size decreases.

Similar to our FAR results, Murly et al. [20] found peroneus longus functioned at a significantly lower activation during the contact and midstance phases of gait in the flat‐feet group when compared to normal‐arched feet [20]. Although not significant in another EMG study, lower activation was found in peroneal muscles in the PFP group when compared to the healthy cohort [21]. FAR is developed to mimic the normalizing strategy similar to EMG studies that use the resting state (relaxed muscle) in the denominator and allows us to better understand the muscle function [16]. This mechanism normalizes the activity of the muscle (during a functional task such as SLS) to the non‐weight‐bearing sidelying position, however, with an extra advantage of visual isolation of the particular muscle under observation, eliminating the chances of cross‐talk [16]. Although not directly related to our results, it's worth noting that besides its role as an evertor of the foot, the peroneus longus, working in conjunction with the abductor hallucis, also plays a crucial role in stabilizing the medial longitudinal arch after heel strike. [22]. It is possible that lower activation or a comparatively lower increase in CSA of the peroneal muscle group in the weight‐bearing position may contributes to the flattening of the arches in PFP patients, which may add to the faulty mechanics (increased pronation) at the distal foot and ankle joints. This further explains the significant inverse correlation between the FPI and muscle CSA of the peroneal muscles in the weight‐bearing SLS functional position established in this study: the greater the foot pronation, the smaller the size of CSA of the peroneal muscle group in individuals with PFP. This delineates a potential functional dysfunction in the peroneal activation in the functional position, which couldn't be seen in the USI assessment performed in the non‐weight‐bearing sidelying position (Figure 2).

Angin et al. [15] found smaller muscle size of peroneal muscles in the pes planus group. This helps explain this study's findings further; we also had a significant negative correlation between the FPI and peroneal muscle size in weight‐bearing, suggesting the more pronated the foot, the smaller the muscle size. Although it is not measured in this study but one potential explanation of it could be that the foot is going into a more pronated position, putting more demand on the invertors of the foot, such as the tibialis posterior, the peroneal muscle group might have diminished activity during functional positions to enhance the activity of supinator muscles [20]. A more dynamic pronation of the foot may result in an increase in dynamic knee valgus, which is linked as a major biomechanical abnormality in individuals with PFP. Contrary to our results, the only other study that investigated muscle size in peroneal muscle in a PFP group [23] found greater muscle thickness in the peroneal muscle group in the PFP group compared to healthy controls. However, it must be noted that all the measurements in this study were performed in the non‐weight‐bearing position, which precludes the ability to understand the ability of the peroneal muscles to activate during a functional stance, such as SLS [23]. We also had a smaller effect size of 0.2 in the non‐weight‐bearing position between the two groups, which increased to 0.73 when the measurements were repeated in the SLS weight‐bearing position, showing a distinct increase in the activation of peroneal muscles in the healthy group that was not observed in the PFP group. Furthermore, there was no attempt made to investigate the foot postural alignment in the PFP group included in this study, which also makes it difficult to assess whether there was a relationship between a particular foot alignment in the PFP group and peroneal muscle size [23].

This holds significant clinical implications for the management of individuals with PFP. There is a need to develop and enhance methods for muscle assessments in dynamic and functional positions that better reflect everyday tasks, as opposed to relying solely on traditional manual muscle testing. Further studies are needed to understand the changes in the muscles distal to the knee joint in individuals with PFP which may provide valuable insight to enhance the rehabilitation programs for individuals with PFP and potentially improve clinical outcomes in this population.

## 5 LIMITATIONS

This study is not without limitations. We imaged the peroneal muscles in the most functional SLS position possible to assess peroneal muscles in the loaded position while maintaining the stance foot on the ground. However, the FAR measures still need further development in USI studies especially for the lower extremity studies. It is imperative to further validate FAR in lower extremity muscles using concurrent EMG studies using different functional positions. Future work should study these muscles in other functional activities/positions, since USI provides us a unique advantage of not only assessing peroneal muscles in the non‐weight‐bearing position, but also in the weight‐bearing functional position. Moreover, assessing other extrinsic lower muscles, including the muscles on the medial leg along with the lateral leg muscles, can provide a more comprehensive understanding of distal muscle adaptations in the PFP group. In addition, this is a cross‐sectional study and thus cannot determine causation. We did not assess relationships to patient‐reported outcomes, so it is unclear if these adaptations contribute to pain/dysfunction. Images were taken in static conditions, thus unclear if these would relate to dynamic function.

## 6 CONCLUSIONS

This is the first study to investigate the peroneal muscle group in the weight‐bearing functional position in a PFP group. The results of this study showed that individuals with PFP had smaller peroneal muscles CSA in the weight‐bearing position with lower FAR when compared to a healthy group. We also found a significant inverse correlation between the FPI score and the peroneal muscle size in SLS, which shows that, with the more pronated foot, the peroneal muscle size decreases. Future studies are needed to determine changes in muscle size and activation ratios because of rehabilitation.

## AUTHOR CONTRIBUTIONS

Abbis Jaffri designed the study. Abbis Jaffri and Andrea Baellow collected and analyzed the data. Abbis Jaffri and Amber Schwarting drafted the manuscript. All authors critically revised the manuscript for important intellectual content. All authors read and approved the final version of the manuscript.

## CONFLICT OF INTEREST STATEMENT

The authors declare that they have no competing interests.

## ETHICS STATEMENT

The study was approved the Institutional Review Board of the University of Virginia.

## CONSENT FOR PUBLICATION

Not applicable.

## Data Availability

Data and materials can be available upon request.
